# Early Assessment of the Invasiveness of the Alien Plant *Vernonia amygdalina* Del. Introduced in China

**DOI:** 10.1002/ece3.73420

**Published:** 2026-04-01

**Authors:** Lei Gao, Jia lin Deng, Shu Ning Liu, Yun Lu Ma

**Affiliations:** ^1^ Guangzhou Key Laboratory of Subtropical Biodiversity and Biomonitoring School of Life Sciences South China Normal University Guangzhou China

**Keywords:** allelopathy, Asteraceae, invasive species, *Vernonia amygdalina*

## Abstract

Invasive species have attracted widespread attention because of their ecological, economic, and social impacts. *Vernonia amygdalina* Del. (commonly known as bitter leaf) is a perennial shrub in the Asteraceae family, native to tropical Africa and widely recognized for its medicinal value. In recent years, this species has been reported sporadically in southern China, raising concerns about its potential to become invasive. Here, we assess the risk of invasion posed by *V. amygdalina* through synthesizing evidence on its taxonomic background, morphological and life‐history traits, reproductive strategies, allelopathic effects, defense mechanisms, climate suitability, and human‐mediated introduction and cultivation practices. Overall, the available evidence suggests that *V. amygdalina* may have substantial invasion potential, supported by high reproductive capacity, strong allelopathic effects, effective defense traits, and broad climatic tolerance. Moreover, current introduction and cultivation practices in southern China may further increase invasion risk by elevating propagule pressure. Preventive actions, including early detection, targeted monitoring, and surveillance systems will be important for managing this species and mitigating potential ecological threats. Our synthesis also highlights the value of assessing invasion risk before introducing non‐native species.

## Graphical abstract

This study evaluates the invasion potential of *Vernonia amygdalina* by synthesizing evidence on its taxonomic background, morphological and life‐history traits, reproductive strategies, allelopathic effects, defense mechanisms, climatic suitability, and human‐mediated introduction and cultivation practices. Overall, the evidence suggests that *V. amygdalina* may have substantial invasion potential, supported by high reproductive capacity, pronounced allelopathic effects, effective defense traits, and broad climatic tolerance.

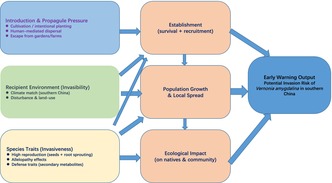

## 1 Introduction

Invasive species are major drivers of global ecosystem change and have received increasing attention (Devenish et al. 2024; Holdrege et al. 2024; Rodrigues et al. 2024), underscoring the need to advance research on invasion mechanisms and control strategies. Studies of biological invasions generally focus on three core components: (i) the invasive potential of species, (ii) the invasibility of recipient environments, and (iii) effective control measures. Environmental invasibility refers to the susceptibility of a habitat to invasion. It is shaped by climate suitability, resource availability, disturbance regimes, and human‐mediated habitat modification, all of which can facilitate the establishment and spread of alien species (Davis et al. 2000; Shea and Chesson 2002). Control measures aim to prevent or limit invasion through early warning, targeted monitoring, and rapid response (e.g., containment or eradication at the incipient stage), thereby reducing ecological impacts and the long‐term management costs (Reaser et al. 2020; Heimovitz et al. 2024). Assessing a species' likelihood of becoming invasive is a prerequisite and a central focus of invasion research, providing essential guidance for management and monitoring strategies (Antunes and Schamp 2017).

Invasive species originate outside their introduced ranges and enter new environments through nonnatural pathways, primarily via human activities, and they can exert substantial negative impacts on native ecosystems (Khatri et al. 2025a; Negi et al. 2025). Once established, they can self‐reproduce and cause ecological, social, and economic harm (Li et al. 2001; Li and Chen 2002). Many invasive plants show vigorous growth and competitive dominance in novel environments (Khatri et al. 2022). Among major threats to global biodiversity, biological invasions rank second only to habitat destruction (Fartyal et al. 2025). In recent decades, international trade and human mobility have accelerated both intentional and accidental introductions of plant species across regions (Negi et al. 2023), contributing to the escalating global invasion problem (Seebens et al. 2017; Khatri et al. 2023). Importantly, not all exotic species become invasive; therefore, evaluating the invasion potential of non‐native species is critical for identifying those that may pose future risks (Li et al. 2001).

Furthermore, China is among the countries most severely affected by biological invasions (Turbelin et al. 2024). Since the 1980s, China has conducted risk assessments for alien species, including evaluating quarantine significance for harmful pests and weeds (Fan and Zhao 1997; Li and Xie 2002). Risk‐assessment systems for invasive species have since been continually refined (Hao et al. 2011; Wan et al. 2017; Zheng et al. 2024). Nevertheless, limitations—including incomplete information on species, insufficient suitability analyses, and challenges in ensuring objectivity—have constrained broad application of these frameworks (Li et al. 2022). Because many traits relevant to invasiveness are difficult to quantify, predicting whether an alien species will become invasive remains challenging. Beyond intrinsic species traits, environmental invasibility and anthropogenic factors often play key roles (Weber and Li 2008). For example, Gao et al. (2018) summarized general patterns of invasive plants in China and the United States by analyzing taxonomic, morphological, and reproductive traits, invasion history, native‐range information, and human influence. Integrating such general invasion‐related traits to evaluate specific non‐native species can support early warning and proactive management (Pfadenhauer and Bradley 2024; Pili et al. 2024).

**FIGURE 1:**
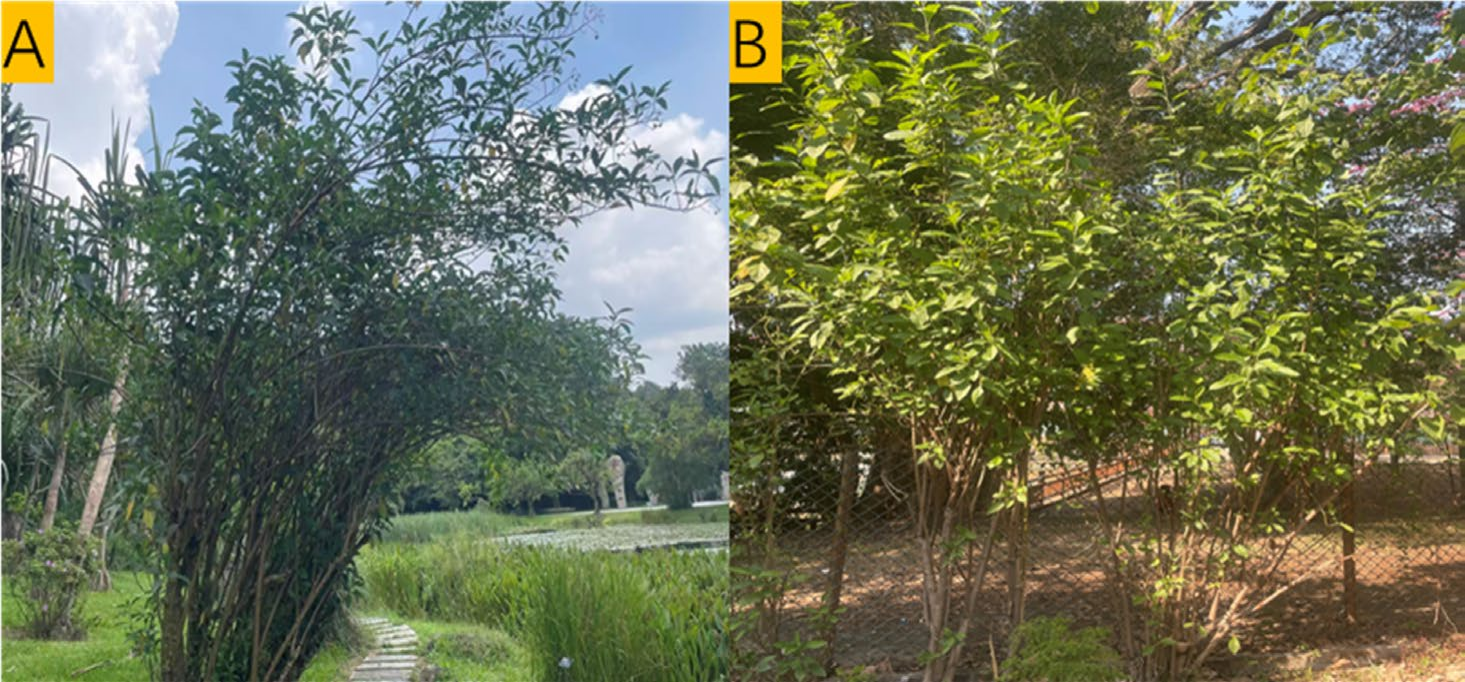
Wild individuals of *Vernonia amygdalina* observed on the campus of South China Normal University (A) and the South China National Botanical Garden (B). The species is capable of natural regeneration and local spread under field conditions; mature plants typically reach ~2–5 m in height. Photographs were taken by the authors.

Building on this framework, we assessed the invasive potential of *Vernonia amygdalina* Del., an alien species that is currently sparsely distributed in southern China (Figure 1). We synthesized available evidence on its invasion‐related traits. Our aim is to develop a conceptual framework for evaluating the invasion potential of alien species. *V. amygdalina* is a perennial shrub or small tree in the Asteraceae family, native to tropical Africa. It has recognized medicinal value in traditional medicine (Alebie et al. 2017) and is widely cultivated in many regions worldwide. In recent years, it has been reported sporadically occurring in southern China (Liu et al. 2022). Based on our review of its traits—particularly taxonomy, distribution, reproductive biology, and potential allelopathic effects—we suggest that *V. amygdalina* may pose a non‐negligible invasion risk in China. This assessment is framed by three working hypotheses: (1) Reproduction hypothesis: *V. amygdalina* may establish and spread readily because it can reproduce efficiently via both seeds and root sprouting. (2) Climate suitability hypothesis: under suitable climatic conditions, *V. amygdalina* may show higher survival and faster expansion. (3) Competitive/allelopathy hypothesis: *V. amygdalina* may gain an advantage over nearby native plants, potentially through allelopathic effects. Overall, this review evaluates the invasion potential of *V. amygdalina* from the perspectives of taxonomy, morphology, reproduction, allelopathy, and defense mechanisms, with the goal of providing early warning of the invasion risk posed by this alien species.

Because explicit studies evaluating the invasiveness of *V. amygdalina* remain scarce—whereas most published work emphasizes its medicinal or forage value—a formal meta‐analysis is not currently feasible. We therefore adopt a qualitative, trait‐based evidence synthesis by systematically collating information from the available literature and complementing it with occurrence records from the Global Biodiversity Information Facility (GBIF) to summarize its distribution pattern. Given the limited number of studies directly testing allelopathy in *V. amygdalina*, we also include a simple seed‐germination bioassay in the allelopathy section to provide empirical support for potential inhibitory effects.

## 2 Taxonomic Characteristics of *V. amygdalina*

*Vernonia amygdalina* belongs to the Asteraceae, one of the largest families of flowering plants and frequently reported among alien and invasive floras. Most Asteraceae species are herbaceous plants, subshrubs, or shrubs, with relatively few tree species. An analysis of the taxonomic traits of invasive plants in China revealed that Asteraceae constitutes a significant proportion of the country's invasive flora, accounting for 23% of invasive species (Gao et al. 2018). Many of the most common invasive species in China belong to this family, including *Solidago canadensis* L., *Pluchea indica* (L.) Less., *Eupatorium adenophorum* Spreng, *Eupatorium odoratum* L., *Bidens pilosa* L., and *Sphagneticola trilobata* (L.) Pruski (Gao et al. 2018).

The invasive species from the family Asteraceae are often characterized by their strong invasiveness and destructive impacts, attracting considerable attention. For instance, Yang et al. (2023) studied 33 invasive Asteraceae plants in China and found that, under current climatic conditions, their potential distribution is primarily concentrated in southeastern China. Many Asteraceae are fast‐growing and produce capitulum inflorescences with numerous small seeds, typically bearing a pappus that facilitates wind dispersal. In addition, allelopathy is common in the family. Together, these traits contribute to the prominence of Asteraceae among invasive plants worldwide. Accordingly, the appearance of alien Asteraceae species in a region warrants heightened vigilance and proactive risk assessment (Yang et al. 2023).

## 3 Morphological Characteristics

In contrast to many other Asteraceae species, *V. amygdalina* is primarily a shrub or small tree, typically 2–5 m tall and occasionally reaching 10 m (Ijeh and Ejike 2011; Houegban et al. 2024). It has a well‐developed root system that is drought‐tolerant and adaptable to a range of soil conditions (Bonsi et al. 1995; Kaur et al. 2019). Roots can sprout upright shoots, enabling clonal propagation (Bonsi et al. 1995).

The bark of *V. amygdalina* is rough, and its stems are robust and highly lignified, providing strong structural support. Stem epidermal cells have thick cell walls and a well‐developed cuticle, which reduces water loss and may facilitate persistence in relatively dry environments (Bonsi et al. 1995).

Capitulum inflorescences are borne terminally or in axils and can produce seeds. The fruit is an achene, cylindrical to top‐shaped with ribs, with a truncate apex often accompanied by callus tissue at the base. Achenes may be short‐haired or glabrous and can sometimes be glandular. The pappus is usually two‐layered, though occasionally single‐layered: the inner layer is elongated and bristle‐like (deciduous or persistent), whereas the outer layer is short, consisting of many or few bristles or scales, and may be absent.

Seeds are small and abundant, conferring high reproductive capacity. Under suitable conditions, this can facilitate rapid dispersal and establishment. Achenes may be dispersed by wind, water, and animals. Seed dispersal efficiency is closely linked to spread, and invasive species often exhibit high dispersal efficiency (Teller et al. 2016; Martín‐Forés et al. 2018). Among dispersal modes, wind dispersal is especially effective (Teller et al. 2016). Work on common invasive Asteraceae in Yunnan, China, shows that dispersal distance is positively related to seed size and pappus traits (Peng et al. 2018). Thus, the small, abundant seeds of *V. amygdalina*, coupled with pappus structures, likely enhance its potential for rapid spread and successful establishment.

## 4 Reproductive Characteristics

The reproductive strategies of *V. amygdalina* are diverse and efficient (Houegban et al. 2024). Its well‐developed root system can produce upright shoots, and field observations suggest it can propagate clonally in the wild (Houegban et al. 2024), potentially forming monoclonal patches. Clonal reproduction is common among invasive plants; for example, 37% of invasive species in the wild rely predominantly on clonal reproduction (Gao et al. 2018). Clonal modes include root sprouting, adventitious roots, stolons, and even propagation via leaves or axillary buds (Gao et al. 2018).

Clonal reproduction can enable rapid occupation of available habitat and secure access to resources. Increases in clonal ramets may also enhance pollen availability, thereby improving sexual reproductive success (Buchanan 2015). Consequently, clonal growth can facilitate successful sexual reproduction (Herrera and Nassar 2009; Mariani et al. 2010; Semizer‐Cuming et al. 2019).

For *V. amygdalina*, sexual reproduction appears to be the primary mode of propagation. Individuals can flower and set seed within the same year. In Africa, seed propagation is the main method used in cultivation (Houegban et al. 2024). Under natural conditions, seeds typically germinate within 2–4 weeks. During the flowering season, individuals can produce large numbers of seeds, potentially allowing rapid occupation of a habitat within a few years after introduction.

## 5 Allelopathic Effects

Allelopathy is widely recognized as a mechanism that can enhance plant competitiveness (Bieberich et al. 2018; Wang et al. 2024; Joshi et al. 2025; Khatri et al. 2025b), and is often framed as a “novel weapon” in plant invasions (Svensson et al. 2013; Zheng et al. 2015; Qi et al. 2020). Trait compilations indicate that 52% of invasive species in China and 36% in the United States show allelopathic capacity (Gao et al. 2018). Many invasive plants release secondary metabolites (e.g., phenolics) into the surrounding environment via leaching, volatilization, and other pathways. These compounds can inhibit germination and growth of neighboring plants and thereby reduce their competitiveness (Kim and Lee 2011; Al Harun et al. 2014; Kato‐Noguchi and Kurniadie 2022).

Allelopathic effects can reduce seed germination of native plants and ultimately lower local biodiversity (Kim and Lee 2011). Leaves of *V. amygdalina* contain abundant phenolic compounds (Farombi and Owoeye 2011). In other systems, strong allelopathy has been linked not only to reduced growth of neighboring plants but also to reduced pollinator visitation and lower sexual reproductive success in native species (Masters and Emery 2015).

However, research on allelopathy in *V. amygdalina* remains limited and has largely focused on its use as a biopesticide (Mkindi et al. 2017; Tembo et al. 2018). However, phytochemical screening of ethanol extracts has identified alkaloids, phenols, flavonoids, terpenoids, saponins, tannins, and cardiac glycosides (Tura et al. 2024), suggesting considerable allelopathic potential that could contribute to invasion success.

**FIGURE 2:**
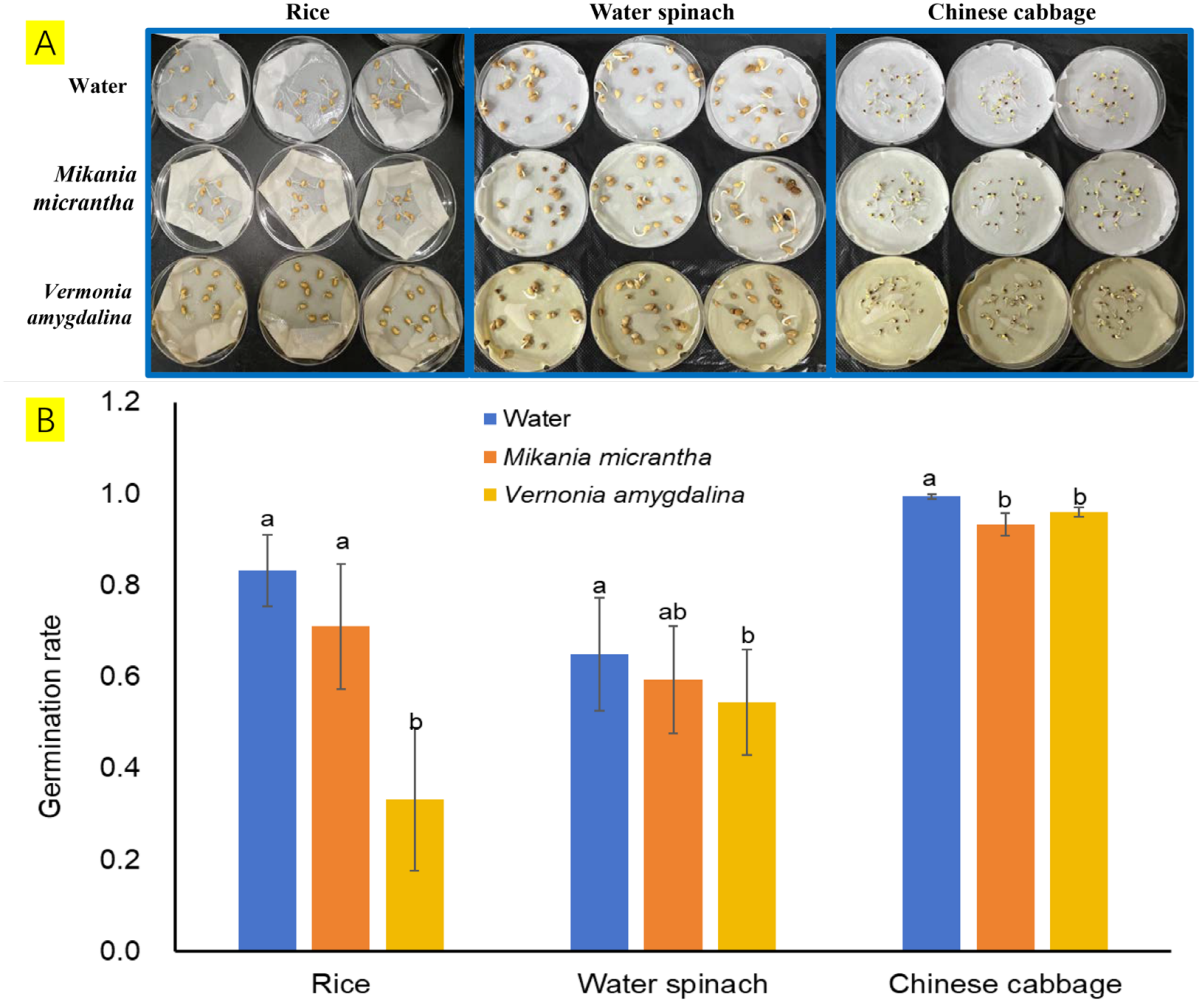
Allelopathic effects of leaf extracts from *Vernonia amygdalina* and the invasive *Mikania micrantha* on seed germination of three crop species. Seeds of rice ( *Oryza sativa* ), water spinach ( *Ipomoea aquatica* ), and Chinese cabbage ( *Brassica rapa* var. *chinensis*) were treated with distilled water (control) or leaf extracts (0.05 g L^−1^) prepared from *M. micrantha* or *V. amygdalina*. (A) Representative photographs taken 3 days after incubation show delayed germination and reduced radicle and plumule growth under the *V. amygdalina* treatment compared with the control and *M. micrantha* treatments. (B) Mean germination rate (±SD) calculated from measurements on days 1, 4, and 7 during a 7‐day observation period. For each crop species and treatment, 60 seeds were used (three replicates). Different letters above bars indicate significant differences among treatments within the same crop species (Duncan's multiple range test, *p* < 0.05; *n* = 9).

To provide empirical evidence, we compared leaf materials from *V. amygdalina* with those from the invasive vine *Mikania micrantha* by applying leaf‐homogenate extracts to seeds of three common crops (rice, water spinach ( *Ipomoea aquatica* Forssk.), and Chinese cabbage ( *Brassica rapa* var. *chinensis*)), using distilled water as a control. By monitoring germination over time, we evaluated whether *V. amygdalina* inhibits germination and early establishment. Leaf extracts of *V. amygdalina* significantly reduced germination vigor of rice and water spinach and markedly inhibited radicle and plumule growth. Overall, the inhibitory effects of *V. amygdalina* on germination and early seedling establishment were stronger than those of *M. micrantha* (Figure 2). *M. micrantha* is widely reported to possess strong allelopathic potential: leaf and/or root extracts and isolated allelochemicals can inhibit germination and seedling growth of multiple recipient species (Wu et al. 2009, 2015; Ma et al. 2020). Together, these findings support the view that *V. amygdalina* may also exhibit pronounced allelopathic effects.

## 6 Plant Defense Mechanisms

In addition to allelopathy, another important mechanism is defense against herbivores. Invasive plants often exhibit effective defense mechanisms. Compared to native species, invasive plants may be attacked less frequently by herbivorous pests. Early work proposed the “enemy release hypothesis,” which suggests that invaders face fewer specialized enemies in introduced habitats (Blossey and Notzold 1995). More recent studies indicate that invasive plants still experience pressure from generalist herbivores (Sun et al. 2023), which may select for enhanced defenses (Zhai et al. 2024).

*V. amygdalina*, commonly called bitter leaf, has a characteristic bitter taste caused by anti‐nutritional compounds such as alkaloids, saponins, tannins, and glycosides (Bonsi et al. 1995; Farombi and Owoeye 2011). In Africa, *V. amygdalina* has been investigated as an alternative insecticide for pest control (Green et al. 2017; Mkindi et al. 2017), indicating strong pest‐defense capacity. In newly introduced areas, reduced herbivore pressure—combined with intrinsic defense traits—could facilitate population growth and spread.

## 7 Climatic Distribution

**FIGURE 3:**
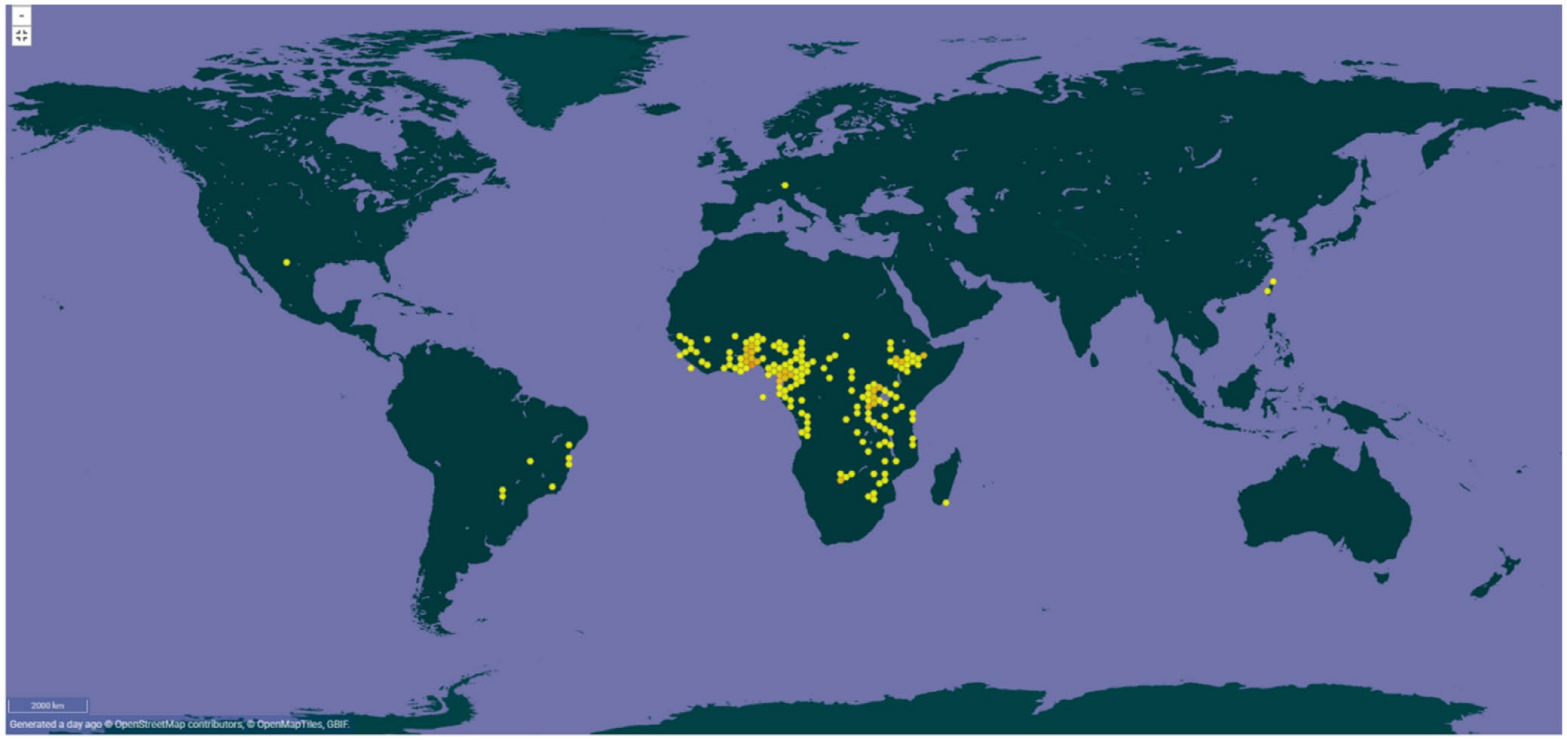
Global distribution of *Vernonia amygdalina* based on GBIF (Global Biodiversity Information Facility) occurrence records. Yellow points represent georeferenced occurrences compiled in the GBIF database, showing a strong concentration in tropical Africa and generally sporadic records elsewhere, including a single recorded region in China (Taiwan) and comparatively more records in Brazil.

*Vernonia amygdalina* occurs naturally across tropical Africa, from East Guinea to Somalia and northeastern South Africa, and it is also reported from Yemen. It is widely cultivated as a vegetable in Benin, Nigeria, Cameroon, Gabon, and the Democratic Republic of Congo, with smaller‐scale cultivation in neighboring regions (Houegban et al. 2024). GBIF (Global Biodiversity Information Facility) occurrence records show a strong concentration of records in tropical Africa, consistent with its native range. Records outside Africa are generally sparse and scattered, with comparatively more occurrences in Brazil. In China, GBIF currently documents the species only from Taiwan (Figure 3). The species typically occurs along riverbanks, lake shores, forest margins, woodlands, and grasslands, in areas with mean annual rainfall of 750–2000 mm and elevations up to 2800 m (Farombi and Owoeye 2011; Houegban et al. 2024). Although drought tolerant, *V. amygdalina* performs best in humid, humus‐rich soils (Kaur et al. 2019; Houegban et al. 2024; Khatri et al. 2025a).

China spans climates from tropical to cold temperate. South of the Yangtze River, the subtropical monsoon zone is characterized by alternating influences of tropical maritime and polar continental air masses. This region has abundant heat resources, with mean annual temperatures of 13°C–20°C and accumulated temperatures above 10°C of 4000°C–6500°C (Sun et al. 2008). In January, mean temperatures south of the Yangtze River generally exceed 0°C (2°C–10°C) and reach 10°C–12°C in southern subtropical areas of Guangdong and Guangxi. Annual precipitation typically ranges from 800 to 1600 mm (Yao et al. 2008). Such climatic similarity to the species' native tropical range may facilitate survival and reproduction in southern China. This similarity may reduce environmental resistance and increase the likelihood of establishment and spread.

## 8 Introduction and Cultivation Practices

*Vernonia amygdalina* has notable medicinal value. Its bitter leaves are described as “cool” in traditional use and are used for detoxification and treatment of several ailments. In China, it is commonly used as a folk remedy for fever and gastrointestinal disorders. Fresh leaves may be eaten raw, decocted, dried and ground into powder, or applied externally (Liu et al. 2022). In addition, leaf extracts have been investigated for roles in targeted cancer therapies (Johnson et al. 2017; Yao et al. 2020; Liu et al. 2022).

In southern China, these perceived benefits have promoted widespread cultivation (Yao et al. 2020; Liu et al. 2022). However, poor management during cultivation and human‐mediated escape into the wild may increase invasion risk. Surrounding habitats near cultivation sites may function as “stepping stones” that facilitate spread. Seeds or vegetative propagules can disperse through natural pathways, expanding the species' range and elevating invasion potential.

**FIGURE 4:**
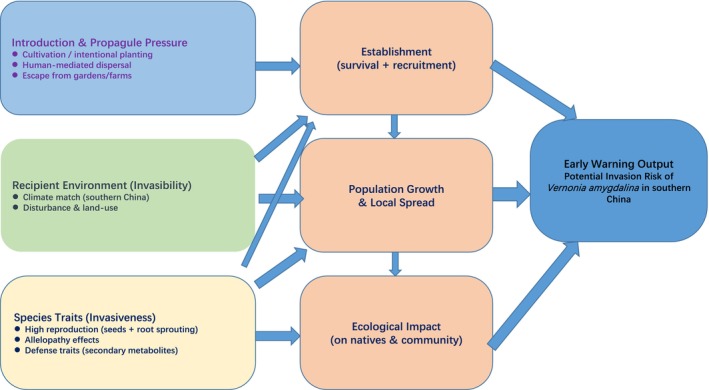
Conceptual framework illustrating the potential invasion of *Vernonia amygdalina* in China. This framework starts from the propagule potential associated with its introduction and cultivation and integrates climatically suitable regions in China with its reproductive, growth, allelopathic, and other traits to indicate its invasion potential in China.

To integrate the diverse lines of evidence in this study and present the logical chain more intuitively, we constructed a conceptual framework (Figure 4) depicting the potential invasion threat posed by *V. amygdalina* in China. The framework begins with propagule pressure associated with introduction and cultivation, and it integrates climatically suitable regions in China with reproductive, growth, allelopathic, and other life‐history traits, thereby indicating invasion potential and providing a basis for subsequent monitoring, early warning, and management (Figure 4).

## 9 Conclusions

Although currently recorded only sporadically in southern China (Peng et al. 2018; Yao et al. 2020; Yang et al. 2023), *V. amygdalina* shows a non‐negligible invasion risk of establishment and spread. In particular, high reproductive capacity (seed production plus vegetative regeneration), potential allelopathic effects, and strong defense traits may facilitate establishment and competitive dominance once the species escapes cultivation. Climatic similarity between southern China and the native tropical range, together with ongoing introduction and cultivation, could further increase propagule pressure and create “stepping‐stone” sites for spread. We therefore recommend precautionary management, including targeted monitoring around cultivation areas, early detection and rapid response to escaped individuals, and clearer guidance on planting and disposal practices, to prevent this species from transitioning from an occasional alien to an established invader.

## Author Contributions

**Lei Gao:** conceptualization (equal), funding acquisition (equal), investigation (equal), writing – original draft (equal), writing – review and editing (equal). **Jia lin Deng:** methodology (equal), software (equal). **Shu Ning Liu:** data curation (equal), writing – original draft (equal). **Yun Lu Ma:** data curation (equal), writing – original draft (equal).

## Funding

This study was supported by the National Natural Science Foundation of China (Grant No. 31470451) and the Basic and Applied Basic Research Foundation of Guangdong Province (Grant No. 2024A1515011114).

## Conflicts of Interest

The authors declare no conflicts of interest.

## Data Availability

No additional datasets were generated or analyzed during the current study. All relevant data are included in this article.
